# Confidence-aware self-supervised learning for dense monocular depth estimation in dynamic laparoscopic scene

**DOI:** 10.1038/s41598-023-42713-x

**Published:** 2023-09-16

**Authors:** Yasuhide Hirohata, Maina Sogabe, Tetsuro Miyazaki, Toshihiro Kawase, Kenji Kawashima

**Affiliations:** 1 The Department of Information Physics and Computing, The University of Tokyo, Tokyo, 113-8656 Japan; 2 The School of Engineering Department of Information and Communication Engineering, Tokyo Denki University, Tokyo, 120-8551 Japan

**Keywords:** Biomedical engineering, Translational research, Computational science

## Abstract

This paper tackles the challenge of accurate depth estimation from monocular laparoscopic images in dynamic surgical environments. The lack of reliable ground truth due to inconsistencies within these images makes this a complex task. Further complicating the learning process is the presence of noise elements like bleeding and smoke. We propose a model learning framework that uses a generic laparoscopic surgery video dataset for training, aimed at achieving precise monocular depth estimation in dynamic surgical settings. The architecture employs binocular disparity confidence information as a self-supervisory signal, along with the disparity information from a stereo laparoscope. Our method ensures robust learning amidst outliers, influenced by tissue deformation, smoke, and surgical instruments, by utilizing a unique loss function. This function adjusts the selection and weighting of depth data for learning based on their given confidence. We trained the model using the Hamlyn Dataset and verified it with Hamlyn Dataset test data and a static dataset. The results show exceptional generalization performance and efficacy for various scene dynamics, laparoscope types, and surgical sites.

## Introduction

The increasing demand for minimally invasive surgery in recent years has lead to the growing popularity of endoscopic surgery including laparoscopic operations^1^. One of the reasons for the widespread use of laparoscopic surgery is the increased sophistication of information provided by laparoscopes. In particular, with the advent of improved stereo laparoscopes and 4K/8K laparoscopes, it has become possible to acquire spatial information from laparoscopic images during surgery and examination. As a result, early detection of minute lesions, more accurate measurement of tumor size, and more precise grasp of the positional relationship between the organ and surgical instruments during surgery are now feasible. Furthermore, the acquired 3D information is expected to be applied to computer-assisted surgery, as typified by robot control support and surgical navigation^2–5^.

Laparoscopes are classified into stereo laparoscopes and monocular laparoscopes according to the number of optical systems at the tip of the scope. The mainstream stereoscopic laparoscope is a stereo laparoscope that incorporates two optical systems at the tip. With a stereo laparoscope, the depth can be estimated by stereo vision from the disparity of the left and right images. Semi-Global block Matching (SGM)^6^ and Efficient LArge-scale Stereo (ELAS)^7^ estimate depth from binocular disparity by performing matching on only one set of image pairs, with the latter method having real-time capability. These methods are able to perform depth estimation from a pair of images that are simultaneously acquired from a stereo camera, and reconstruct dense depth information even if the images are time-varying with deformation due to organ excision or grasping. Particularly in the in vivo depth estimation task, ELAS is a typical method that has been substituted as baseline and the ground truth of depth^8–12^. The accuracy of these stereo depth estimation methods depends on the accuracy of pixel-by-pixel matching. Intensity correlation was conventionally employed for the matching, but in recent years, a learning-based matching method using Deep Neural Network (DNN) has been proposed, providing improved accuracy^13–15^. However, a stereo laparoscope requires two optical systems incorporated into the tip of the scope, increasing the scope diameter and cost, so that a monocular laparoscope may be preferred in some cases and depth estimation should be necessary.

For a monocular laparoscope, depth estimation could be performed from multiple monocular images. One example method acquires two images while measuring the position and rotational posture to perform pseudo-stereo-like depth estimation^16^. Other methods such as Structure from Motion (SfM)^17^ and Visual Simultaneous Localization and Mapping (Visual SLAM)^18^ estimate depth with monocular images obtained from multiple viewpoints. However, a pseudo-stereo-like depth estimation method has a limit in the imaging speed because a physical acquisition of two images is inherently required. This limitation causes a decrease in frame per second (fps), so that its application to operation may be difficult where dynamic change is large. In addition, while multi-viewpoint depth estimation methods such as SfM and Visual SLAM estimate depth by reconstructing the camera’s posture and the three-dimensional structure of the feature points from the feature points of the image, the dynamic image scene practically obtained within the living body may provide only a scarce and uniform texture compared to general natural images, resulting in very non-uniform and sparse 3D reconstructed structure. Moreover, in actual surgery, the accuracy of reconstruction may be significantly deteriorated because the entire surgical field is constantly changing due to the autonomous movement of an organ itself and the deformation of an organ due to contact with surgical instruments such as forceps. Therefore, in order to establish a monocular depth estimation method that is effective even in intraoperative operations where large dynamic changes occur, a learning-based method from a single monocular laparoscopic image from one viewpoint is advantageous. However, although monocular depth estimation for urban and natural images has been actively studied, the realization of monocular depth estimation for clinical applications to computer-assisted surgery, such as surgical navigation in actual surgical environments, is still a challenging and unsolved problem. In particular, there is a significant problem in learning-based monocular depth estimation using an laparoscope for surgical applications. Although it is desirable to train monocular depth estimation models for surgical applications using actual surgical scenes, actual surgical scenes contain various deformations, smoke, and other artifacts, which may generate unreliable supervisory signals and may not yield desirable models. However, to the best of our knowledge, no learning architecture has yet been proposed for monocular depth estimation of dynamic laparoscopic scenes that takes into account the reliability of the supervisory signal.

In light of the existing challenges, this study aims to enhance learning and estimation accuracy within dynamic laparoscopic settings, with a particular emphasis on surgical navigation applications. One of the primary obstacles is the acquisition of a training dataset that accurately represents the real surgical environment. However, genuine surgical images contain substantial noise, making it difficult to establish an accurate ground truth.

To address this issue, we introduce a confidence level for the monitoring signal and propose a confidence-aware learning architecture that allows the utilization of real laparoscopic surgical images as the training dataset. This approach aims to adapt to dynamic laparoscopic scenarios characterized by continuous changes in organ deformation, bleeding, and laparoscope positioning.

This paper presents depth estimation for dynamic monocular laparoscopic scenes using the Hamlyn Dataset^8^ and evaluates its performance, resulting in an improved overall depth estimation accuracy independent of target motion. The primary contributions of the proposed method include:

- A platform that allows for depth estimation in the actual surgical field, where obtaining datasets with accurate ground truth is challenging, and enables the acquisition of indirect depth information from stereo endoscopic images during real surgery without requiring precise depth information from Lidar or other sources to be used as a training dataset.
- mechanism to handle irregularities such as noise in the training dataset by taking not only the depth information but also its confidence as input to enable the selection and weighting of the monitoring signal.
- We propose a monocular depth estimation model learning architecture that employs a loss function and a stereo vision-based self-monitoring signal module, which outputs disparity and its confidence level. The monocular depth estimation model trained in this manner facilitates learning in dynamic laparoscopic scenes and single-frame monocular depth estimation. This capability is crucial in laparoscopic surgery, where the endoscopic camera is frequently repositioned based on the operating field conditions.

In the field of computer vision, the recent development of DNN has promoted research on learning-based monocular depth estimation methods, with many application examples of urban scenes. Also in the laparoscopic scene, there have been several studies on depth estimation from monocular images. Ye et al.^8^ proposed a self-supervised learning framework for a monocular depth estimation Convolutional Neural Network (CNN) model, which is an improved version of DeConvNet^19^. They used the disparity information obtained by a classical stereo algorithm from a stereo image pair as a self-supervisory signal. This was one of the earliest methods developed for monocular depth estimation by DNN for the laparoscopic scene.

The latest research by Liu et al.^20^ has proposed a learning method using the sparse three-dimensional structure obtained from SfM and the camera attitude as supervisory signals. Since this method’s learning depends on the accuracy of the SfM algorithm, the accuracy of SfM may decrease and the possibility that correct learning is not performed may increase if training data that involves dynamic deformation of the entire surgical field due to the deformation of a living body itself and interaction with forceps is used for learning in the actual intraoperative laparoscopic scene.

Recently Zhou et al.^21^ have proposed a self-supervised learning framework using monocular image sequences for monocular depth estimation tasks in urban scenes. In this method, self-supervised learning of a monocular depth estimation model by monocular image sequences is realized by introducing explainability mask which handles edge cases such as object movement and occlusion while training PoseNet together with DepthNet at the same time. This method is called SfMLearner and several extensions have been made by warping the features^22^ and introducing a per-pixel minimum reprojection loss and auto-masking process that removes stationary pixels^23^. However, according to the latest research by Shao et al.^24^, the light source often moves in the endoscopic scene, which is very unlikely in the urban scene, so that the light source conditions between images do not match. Moreover, the surface of a living body has small feature amount and strong non-Lambertian reflection may be also generated. It was confirmed that the performance of the conventional SfMLearner, which was aimed for urban scenes, deteriorates under these poor conditions. To improve the performance of SfMLearner for endoscopic scenes, Shao et al.^24^ extended SfMLearner by introducing appearance flow to align the brightness conditions between frames and adopting a feature scaling module to refine the feature expression, and successfully adapted the method to the endoscopic scene.

These methods are extremely effective for applications such as endoscopic diagnosis of static sites. However, there are problems with its application to dynamic surgical scenes. The conventional methods have been designed on the premise that most of the images are from static tissues which do not include deformation from interaction with forceps, so that learning with a stationary and clear dataset may be possible. Meanwhile, laparoscopic images obtained during actual surgery contain deformation of an organ itself, spatial changes in objects due to bleeding and excision, and dynamic deformation and artifacts due to interaction with forceps. If we consider applications in actual dynamic surgical scenes, image sequences obtained from actual surgical scenes may be desirable for developing an estimation model. However, data available from surgery are often noisy and less accurate than those obtained in a static environment. Therefore, to realize learning using data from surgical scenes, a novel architecture is required where learning can be performed without being affected by dynamic deformation of an object reflected in the dataset, and can be controlled by selecting/removing and weighting the information based on its accuracy.

Meanwhile, in the field of computer vision where CNN has been the method of choice, a new technique called Vision Transformer (ViT)^25^ has been recently introduced to show similar or better performance compared with the state of the art methods. ViT was derived from Transformer^26^, which was quite successful in natural language processing. In particular, the Dense Prediction Transformer (DPT)^27^ was proposed as a dense monocular depth estimation architecture that uses ViT as the backbone, and exhibits performance equal to or better than the conventional monocular depth estimation model that employs CNN as its backbone. Since ViT has a higher globality than CNN, it is expected to show superior performance in monocular depth estimation in the laparoscopic scene where detailed and consistent estimation of the entire image is required. To the best of our knowledge, there are still no cases where ViT has been adopted as the backbone of the monocular depth estimation model in the laparoscopic scene.

Since application in surgical scenes is assumed where the estimation model has to cope with dynamic laparoscopic scenes distorted by various deformations and artifacts such as smoke, it would be practical to provide supervisory signals as stereo vision for training a monocular depth estimation model that does not depend on the dynamics of an object. However, the depth estimation using the conventional stereo vision only provides disparity prediction without any consideration of its confidence information. Therefore, it was difficult to flexibly handle outliers and select measurement results depending on their confidence level. Consequently, outliers and inaccurate signals were undesirably included in supervisory signals, prohibiting learning and leading to a poor estimation model in some cases. However, a stereo matching method that provides confidence information as well as disparity has been recently proposed^28,29^. Particularly, STereo TRansformer (STTR)^29^ provides reliability of prediction while improving accuracy by employing ViT for matching. It has shown high accuracy results for SCARED^30^, which is a dataset of the medical scene from laparoscopic surgery. Such advancement in stereo vision has made it possible to measure ground truth with higher accuracy than before even in dynamic laparoscopic scenes by appropriately filtering out outliers according to confidence information. It has also enabled the generation of supervisory signals which incorporate not only depth information but also its confidence. This suggests that not only can high-precision disparity be used as a learning resource for the monocular depth estimation model, but also confidence of prediction can be used as the learning resource. As a result, even in the learning of dynamic laparoscopic scenes where outliers are likely to be included in depth supervisory signals, robust and efficient learning can be performed to provide high accuracy estimation if signals are processed appropriately according to the confidence information.

However, conventional architectures that utilize only depth information as a supervisory signal still do not consider its uncertainty, failing to make the best use of the confidence information. Moreover, a learning architecture for a monocular depth estimation model that takes advantage of the confidence information of the supervisory signal is yet to be proposed in the monocular depth estimation task for the laparoscopic scene.

## Methods

In learning methods for monocular depth estimation models that simultaneously learn scale and shift parameters, such as those used in previous studies focusing on endoscopy, overfitting occurs due to biases in the training data caused by camera-specific characteristics and the shooting environment used to generate the data^31^. Therefore, various scale and shift-invariant losses have been introduced in recent studies and the state of the art performance on monocular depth estimation is achieved^32^. Specifically, acquiring scale-shift-invariant models is crucial for monocular depth estimation in dynamic settings like real laparoscopic surgery, offering two primary advantages in clinical applications.

Firstly, these models can be trained on diverse datasets featuring various organs, surgical techniques, and endoscopic cameras. The normalization of relative depth features in relation to scale and shift allows for training on a combination of in vivo datasets from different environments, resulting in a versatile monocular depth estimation model. Furthermore, the capacity to utilize a mix of datasets as training data enables the effective use of in vivo datasets, which are typically scarce and challenging to obtain, thus expanding the dataset.

Secondly, the distance between the laparoscope and the subject constantly changes in actual laparoscopic surgical settings, causing significant variations in scale and shift parameters. The scale and shift values of objects in the operating field differ between surgeries and over time, leading to substantial deviations from the training data. For instance, when suturing blood vessels, the suture area is the focal point, whereas when handling organs or navigating the surgical field, the entire abdominal cavity is viewed from a narrowed perspective to avoid instrument contact with other organs and potential damage. Therefore, learning scale and shift parameters considerably reduce generalizability, even more so than depth estimation for landscapes and buildings, as seen in autonomous operations. Consequently, it is vital to learn relative depth features independent of scale and shift parameters and develop scale- and shift-invariant monocular depth estimation models for various surgical procedures.

Therefore, we proposes a scale and shift-invariant loss function that takes into account the confidence of self-supervisory signals to appropriately learn relative depth information in a manner independent of the scale and shift parameters, and learns it on the assumption that a flow to restore the estimated relative depth to absolute depth will be applied later. This method enables us to obtain a monocular depth estimation model suitable for application in surgical environments.

**Figure 1:**
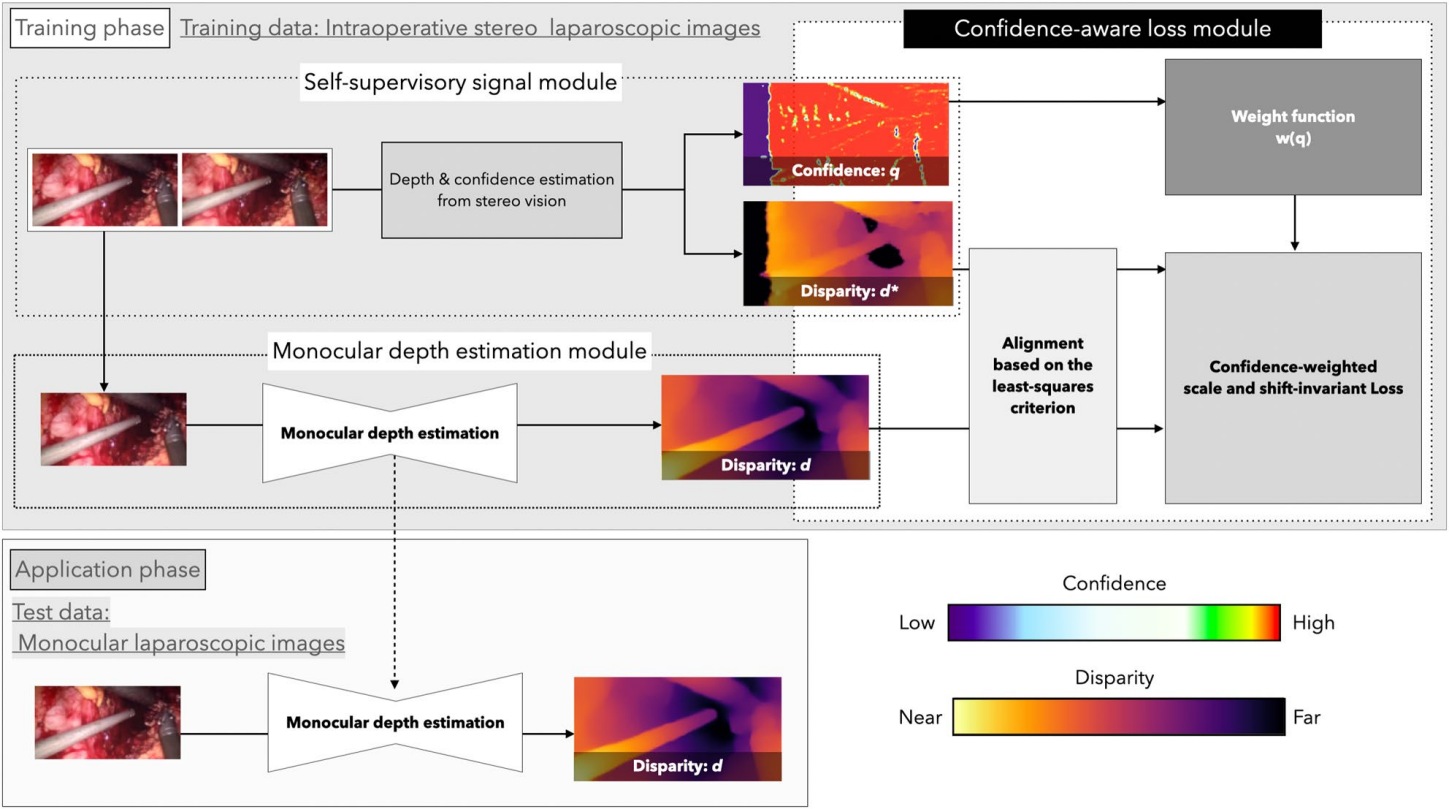
The proposed network architecture. The network in the training phase (top) consists of a stereo vision-based self-supervisory signal module that provides disparity and its confidence as self-supervisory signals, a monocular depth estimation module that actually performs the learning, and a newly designed scale and shift-invariant loss that takes disparity and its confidence as input. In the application phase (bottom), monocular depth estimation is performed using only the learned monocular depth estimation module with a single image as input.

In the following, we describe a method for learning monocular depth estimation models using disparity and its confidence estimated from stereo laparoscope images as self-supervisory signals in order to achieve effective learning in dynamic laparoscope scenes and to achieve dense monocular depth estimation from a single laparoscope image. The overall training architecture is shown in Fig. 1. It consists of a monocular depth estimation model that actually does the training, a self-supervisory signal module composed of stereo vision that outputs disparity and its confidence, and a confidence-aware loss module that includes a scale and shift-invariant loss with disparity and its confidence as input.

### Confidence-aware loss module

#### Confidence

The self-supervisory signal module outputs the estimated disparity and corresponding confidence from a pair of stereo laparoscope images as a real number between 0 and 1. In the following, confidence are denoted by $$q\in \left[ 0,1\right] \ $$. Here, disparity is calculated for every pixel in the training data image, ultimately yielding a disparity and confidence pair for each pixel.

#### Confidence-weighted scale and shift-invariant loss

In this section, we extend the scale and shift-invariant loss proposed in Ranftl et al.^33^ and propose a loss weighted according to the confidence of the self-supervisory signal. First, let *M* be the number of pixels in the image that have valid ground truth, and let $\theta$ be a parameter of the prediction model. Let $d=d(\theta )\in {\mathbb {R}}^M$ be the estimated disparity and $d^*\in {\mathbb {R}}^M$ be the ground truth of the corresponding estimation. To define a scale and shift-invariant loss, we first need to properly fit the scale and shift of the predictions and ground truth. The alignment of the scale and shift between the predictions and ground truth is then performed based on a least-squares criterion:

1 $$\begin{aligned}{} & {} \left( s,t\right) =\mathop {\text {arg min}}\limits _{s,t}{\sum _{i=1}^{M}{(s{d_i}+t-d_i^*)}^2}, \end{aligned}$$

2 $$\begin{aligned}{} & {} {\hat{d}}_i=s{d_i}+t,\ \ {{\hat{d}}_i^*}={d_i^*}, \end{aligned}$$

where $s\in {\mathbb {R}}_+$ is the scale parameter, $t\in {\mathbb {R}}$ is the shift parameter, and ${\hat{d}}_i, {\hat{d}}_i^*\in {\mathbb {R}}$ are the aligned prediction and ground truth. The value corresponding to each pixel *i* is denoted by the subscript *i*. Then, with $w\left( q\right)$ as the weight corresponding to the confidence *q* of the self-supervisory signal of the pixel obtained from the self-supervisory signal module, we define the confidence-weighted scale and shift-invariant loss $L_{cwssimse}$ for a single image as

3 $$\begin{aligned} L_{cwssimse}\left( \hat{{\textbf{d}}},{\hat{{\textbf{d}}}}^*,{\textbf{q}}\right)&=\frac{1}{2M}\sum _{i=1}^{M}w_i(q_i)\left\Vert {\hat{d}}_i-{\hat{d}}_i^*\right\Vert ^2, \end{aligned}$$

where $\hat{{\textbf{d}}}, \hat{{\textbf{d}}}^*, {\textbf{q}}$ are define as $\hat{{\textbf{d}}} = ({\hat{d}}_1,{\hat{d}}_2,\ldots ,{\hat{d}}_M)^{T}, \hat{{\textbf{d}}}^*= ({\hat{d}}_1^*,{\hat{d}}_2^*,\ldots ,{\hat{d}}_M^*)^{T}, {\textbf{q}} = ({q_1},{q_2},\ldots ,{q_M})^{T}$ to represent the inputs collectively.

Furthermore, we weight the multi-scale shift-invariant gradient matching term^33^ adapted to the disparity space according to the confidence and define a new gradient matching term $L_{cwreg}$:

4 $$\begin{aligned} L_{cwreg}\left( \hat{{\textbf{d}}},{\hat{{\textbf{d}}}}^*,{\textbf{q}}\right)&=\frac{1}{M}\sum _{k=1}^{K}\sum _{i=1}^{M}{w_i(q_i)\left( \left| \mathrm {\nabla }_xR_i^k\right| +\left| \mathrm {\nabla }_yR_i^k\right| \right) }, \end{aligned}$$

5 $$\begin{aligned} R_i^k&={\hat{d}}_i-{\hat{d}}_i^*\ (k: scale\ level), \end{aligned}$$

where *k* denotes the scale level at which the image resolution is halved for each level, and $R_i^k$ is the difference in disparity at each scale *k* for pixel *i*. There are *K* scale levels and the value of *K* is set to $K=4$ as in^33^. From the above, the final loss $L_{cwssi}$ for the training set *l* is

6 $$\begin{aligned} \begin{aligned} L_{cwssi}&= \frac{1}{N_l}\sum _{n=1}^{N_l}{L_{cwssimse}\left( {\hat{{\textbf{d}}}}^{n},{\hat{{\textbf{d}}}}^{*n},{\textbf{q}}^{n}\right) } \\&\quad + \alpha \frac{1}{N_l}\sum _{n=1}^{N_l}{L_{cwreg}\left( {\hat{{\textbf{d}}}}^{n},{\hat{{\textbf{d}}}}^{*n},{\textbf{q}}^{n}\right) }, \end{aligned} \end{aligned}$$

where ${\hat{{\textbf{d}}}}^{n}, {\hat{{\textbf{d}}}}^{*n}, {\textbf{q}}^{n}$ denotes ${\hat{{\textbf{d}}}}, {\hat{{\textbf{d}}}}^{*}, {\textbf{q}}$ in each image set *n*, respectively, $N_l$ is the size of the training set and $\alpha$ is set to 0.5.

### Weight functions

In this section, we propose two types of weight functions $w\left( q\right)$.

#### Hard mask

Under the design concept that self-supervisory signals with confidence below a certain threshold $$\theta \in \left[ 0,1\right] \ $$ are ignored as input, those above $\theta$ are input to the loss function as uniform information for learning, we define the weight $w\left( q\right)$ as

7 $$\begin{aligned} w(q) = {\left\{ \begin{array}{ll} 1 &{}\ (\theta \le q \le 1)\\ 0 &{}\ (0 \le q < \theta ) . \end{array}\right. } \end{aligned}$$

In this paper, this weight function is defined as Hard Mask.

#### Soft mask

Under the design concept that self-supervisory signals with confidence below a certain threshold $\theta$ are ignored as input, and those above $\theta$ are input to the loss function as information with importance according to the confidence for learning, we define the weight $w\left( q\right)$ as

8 $$\begin{aligned} w(q) = {\left\{ \begin{array}{ll} e^{\lambda \left( q-1\right) } &{}\ (\theta \le q \le 1)\\ 0 &{}\ (0 \le q < \theta ) , \end{array}\right. } \end{aligned}$$

where $\lambda \in {\mathbb {R}}_+$ is a hyperparameter. The weights are designed so that the higher the confidence of the self-supervisory signals, the larger the weights. The weights are weighted against the squared error between the predictions and the self-supervisory signal, and are designed to make learning more efficient by making it easier to capture accurate features. On the other hand, the weights are also designed to improve robustness by including features with low confidence in the self-supervisory signal, such as artifact-ridden areas, by giving them small weights rather than excluding them entirely. In this paper, this weight function is defined as Soft Mask.

### Monocular depth estimation module

This section describes the details of the monocular depth estimation module. In the monocular depth estimation module, DPT is used as the monocular depth estimation model and trained with a self-supervisory signal provided by the self-supervisory signal module. By using ViT as the encoder, DPT has a higher globality than other monocular depth estimation models that use CNN as the encoder, and achieves detailed and consistent depth estimation across the entire image. Because it is particularly important to achieve detailed and consistent depth estimation across the entire image in dense depth estimation for laparoscopic scenes, DPT is used as the monocular depth estimation model. DPT provides depth predictions in inverse depth space. In this paper, we employ DPT-Hybrid^27^ as the encoder of DPT. Stereo in vivo images obtained from laparoscopes during surgery and examinations are generally small in number, which reduces the number of training data that can be used for fine tuning. For this reason, we employ DPT-Hybrid, which has shown significant performance in fine tuning on small data sets.

### Self-supervisory signal module based on stereo vision with confidence

This section describes a self-supervisory signal module based on stereo vision that provides self-supervisory signals with confidence. It is practical to use stereo vision as supervisory signal provider to train a monocular depth estimation model in a dynamic laparoscopic scene.That is because, in principle, stereo vision is independent of object motion. However, in general, since the object is often close to the laparoscope, the disparity between stereo laparoscope images is large, and matching over a wide disparity range is required for depth estimation by stereo vision. In addition, the accuracy of some stereo vision depth estimation results may deteriorate or invalid disparity may be output due to the possibility of occlusion areas and artifacts such as smoke in intraoperative laparoscopic images. Therefore, in order to use the disparity obtained from stereo images as a self-supervisory signal, it is an important issue to obtain the confidence corresponding to the estimated disparity in order to appropriately weight or exclude those with low accuracy in consideration of the accuracy of the estimation.

**Figure 2:**
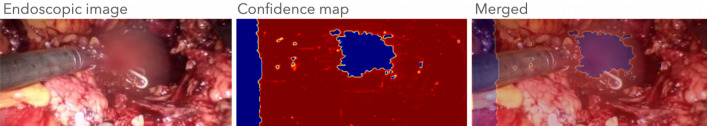
Visualization of confidence score. The outer diameter of fenestrated bipolar forceps is approximately 8.5 mm. The region with confidence 0 on the left side of the confidence map is the region where the disparity was not calculated because the input image is a left-eye image and therefore does not overlap with the right-eye image.

Based on the above, this paper employs STTR as a self-supervisory signal module. Unlike conventional stereo depth estimation methods that only use similarities between pixel intensities as matching criteria, STTR estimates disparity by matching based on the attention calculated from ViT, which alleviates the disadvantage of conventional methods in that it is limited to a fixed disparity range. In fact, STTR has shown high accuracy on SCARED^30^, a medical scene dataset for laparoscopic surgery, which requires recognition of nearby objects^29^. In addition, STTR matches pixels explicitly and densely while imposing a uniqueness constraint, and provides identifies occlusion regions and confidence. Then, it is possible to weight the disparity estimation results appropriately according to the confidence and to exclude those with low accuracy. Therefore, the output of STTR with the estimated disparity and confidence can be used to generate a self-supervisory signal that improves learning performance. In this method, we use the disparity, in other words, the depth predictions in the inverse depth space and confidence from STTR as self-supervisory signals to learn a monocular depth estimation model. In this method, disparity, or depth prediction in inverse depth space, and confidence from STTR are employed as self-supervised signals to train the monocular depth estimation model. Figures 2 and S1 display examples of endoscopic images with diminishing confidence levels and their corresponding confidence maps. The confidence level for water droplets is notably low. This low confidence level pertains to the left side of the left and right stereo images. The confidence level is reduced because regions absent in the right image, specifically pixels on the left side of the left image, cannot be matched and are deemed occlusions. The confidence level of water droplets is remarkably low, almost negligible. This low confidence level pertains to the left-side image of the left and right stereo images. Areas not present in the right-side image, specifically pixels on the left side of the left-side image, cannot be matched and are deemed occluded, resulting in low confidence levels.

### Performance metrics

In this study, we use the following general scale and shift-invariant metrics to evaluate depth predictions. Let the index of each depth predictions be $i = 1,2,...,N$. We use the average absolute value of the relative error (Abs. Rel.) and the Threshold Accuracy as the two metrics, where Abs. Rel. is expressed as $\frac{1}{N}\sum _{i=1}^{N}\frac{\left\Vert d_i-d_i^*\right\Vert }{d_i^*}$, and the Threshold Accuracy is defined by the ratio of pixels whose depth predictions and ground truth satisfy $\max {\left( \frac{d_i}{d_i^*},\ \frac{d_i^*}{d_i}\right) }=\delta <t,\ \ t\in \left[ 1.25,\ {1.25}^2,{1.25}^3\right]$ for each threshold *t*^20,34^. The smaller the value of Abs. Rel. and the larger the value of Threshold Accuracy, the better the performance. Prior to error evaluation, the depth predictions and ground truth scales and shifts are aligned for each image. This alignment is done in inverse depth space based on robust linear regression for outliers. Iteratively Reweighted Least Squares (IRLS)^35^ was used as the algorithm, and the biweight function^36^ was employed as the weight function. The tuning constant was fixed at 4.685, which gives a coefficient estimate with asymptotic efficiency of about 95% for the normal distribution^35^. This evaluation is performed only for pixel pairs with a valid depth.

### Datasets

#### SCARED

SCARED^30^ offers 45 stereo laparoscopic images captured with a da Vinci Xi within a pig’s abdominal cavity immediately post-euthanasia, along with corresponding ground truth 3D point cloud information for each image and 45 stereo videos recorded at 25 fps. Both images boast a resolution of 1280 × 1024 per eye, and the depth ground truth for each pixel is determined through high-precision active stereo vision. Therefore, obtaining clean image data and depth ground truth without disruptions like SCARED dataset necessitates establishing a stringent imaging environment, complicating the data acquisition.

Of the 45 datasets, 35 are designated for training and 10 for testing. The SCARED laparoscopic images are static, without tissue deformation or surgical instrument movement.

#### Hamlyn Dataset: The da Vinci laparoscopic dataset from the Hamlyn Center for Robotic Surgery

This dataset consists of 41,431 pairs of stereo-parallelised stereo images collected during a partial human nephrectomy performed with the da Vinci Si^8^. Of these, 34,240 pairs are provided as training data and 7191 pairs as test data. The resolution of the images is 384 × 192. In this paper, we refer to it as the Hamlyn Dataset. The Hamlyn Dataset contains artefacts such as interaction between the organism and forceps, deformation of the surgical field, smoke, etc., and ground truth by LiDAR, etc., is not provided. In addition to the practical difficulty of providing exact ground truth for image sets in which the surgical field is dynamically deformed^37^. In laparoscopic surgery, which is a typical minimally invasive surgery, it is not possible to open a new port and insert multiple cameras and LiDAR sensors for ground truth acquisition. In addition, during surgery, the object of 3D shape measurement is constantly deformed due to organ resection and suturing, and is also affected by the adhesion of liquid components such as blood and smoke such as water vapour. Therefore, 3D shape measurement using current LiDAR sensors is not realistic. For the same reason, estimation by SfM can also be difficult.

**Figure 3:**
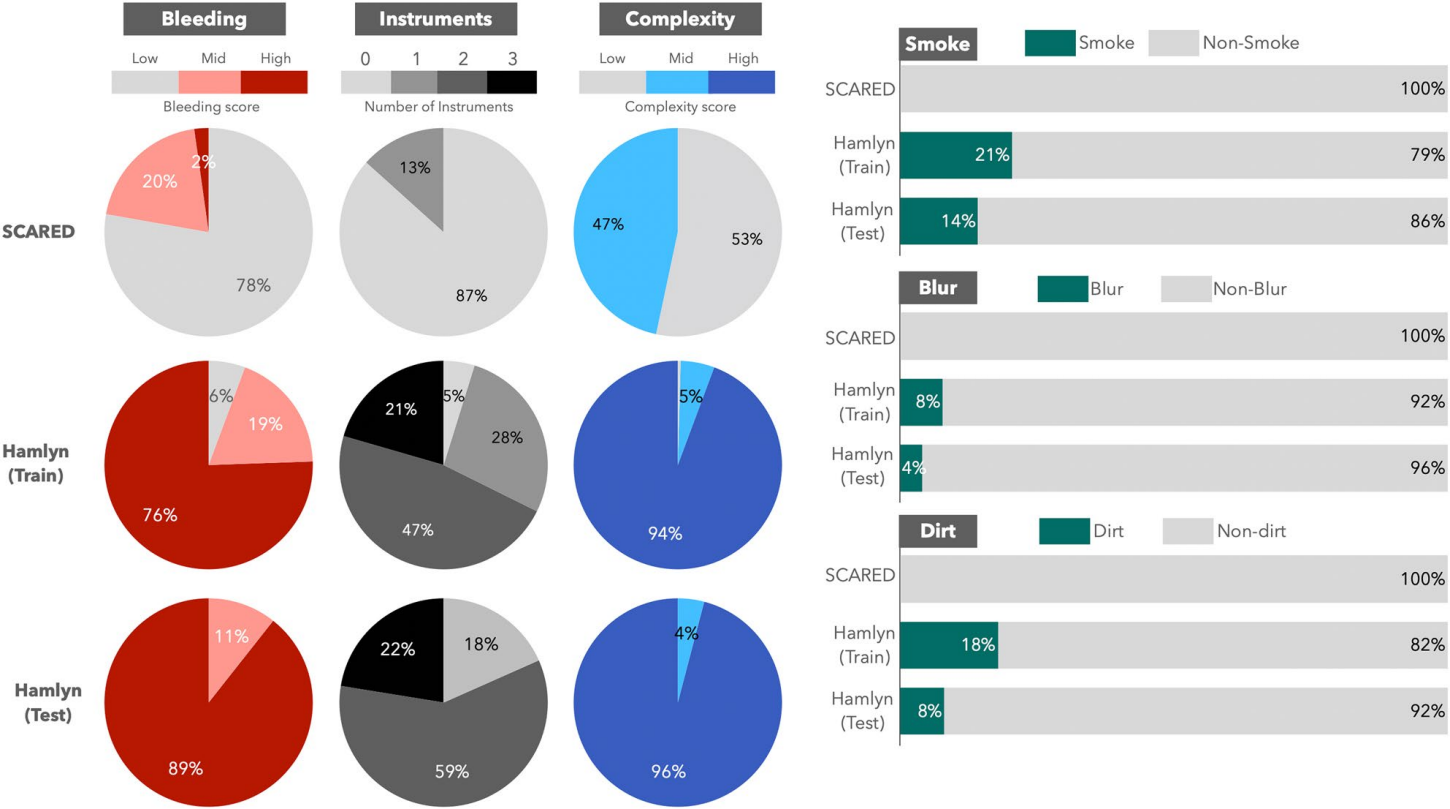
Differences between the Hamlyn and SCARED datasets: the datasets were assessed based on the degree of bleeding (roughly the amount of bleeding in the image), the number of instruments visible in the image, and the complexity of the operative field (Low: images with 2–3 organs, Mid: images with light bleeding and multiple organs and tissues, High: images affected by resection and bleeding). Additionally, the percentage of images containing blur, smoke, and dirt was evaluated.

Figure 3 illustrates the difference in information content between SCARED, a representative dataset with accurate disparity, and the Hamlyn Dataset, consisting of actual stereo endoscopic images. SCARED represents the entire series provided (45 series), while the Hamlyn Dataset comprises 278 images (49 test images and 229 training images), extracted from every 150 images. The complexity of conditions is assessed based on factors such as bleeding, the number of instruments in the image, the presence of smoke, dirt, blurring, resection, and more. The results reveal that actual endoscopic surgical images encompass numerous situations not represented by datasets like SCARED, which provide accurate ground truth. For efficient dataset collection, utilizing common surgical endoscopic images, such as those represented by Hamlyn, as a training dataset will be beneficial for enhancing accuracy and adapting to new algorithms in the future. However, as previously mentioned, obtaining accurate ground truth for clinical surgical images like Hamlyn is challenging. In particular, conventional depth estimation methods such as STTR alone cannot provide sufficient depth estimation due to smoke, contamination and optical distortion of the endoscope itself.

For these reasons, this study designs a framework called Hamlyn Dataset, which uses actual surgical videos as a training dataset. Specifically, the depth calculated using STTR from 7191 pairs of provided test sequence images is treated as a indirect-ground truth and used for evaluation. At the same time, the confidence level is introduced with a weighting function to prevent false learning due to uncertain depth information.

#### Relevance of treating depth predictions obtained from STTR as indirect ground truth

In this section, we verify the validity of treating the depth predictions obtained from STTR as indirect ground truth. It was customary that in vivo depth baselines and indirect-ground truths were often given by ELAS based on stereo images^8–12^. However, laparoscopes that are generally intended to project objects at short distances have a large binocular disparity, which sometimes violate restrictions on the fixed disparity range in the algorithm, leaving some proportion of pixels unmatched and their depth uncalculated. On the other hand, with STTR, the restrictions on the fixed disparity range are relaxed, so matching in a wider range is possible. We also quantitatively compared the estimation accuracy of ELAS and STTR. Of the 45 datasets provided by SCARED, the depth was predicted by STTR for those datasets for which the entire image was properly matched when LIBELAS (Library for Efficient Large-scale Stereo Matching)^7^, which is the original implementation of ELAS, was applied and the depth prediction was done for the entire field of view. The depth estimation outcomes from ELAS and STTR were quantitatively assessed against the ground truth. The findings are displayed in the Table 1. The results indicate that STTR is more consistent with the ground truth compared to ELAS. In addition, more than 99% of the depth predictions of STTR satisfy $\max {\left( \frac{d_i}{d_i^*},\ \frac{d_i^*}{d_i}\right) }=\delta <1.25$ against the ground truth. Together with the result that Abs. Rel. is also a small value of 0.034, it can be safely said that there is no large deviation from the ground truth, so treating the depth predictions obtained from STTR as indirect ground truth may be justified.

**Table 1:** Performance evaluation of STTR and ELAS depth predictions for SCARED.

Method	Abs. Rel.	$\delta <1.25$	$\delta <1.25^2$	$\delta <1.25^3$
ELAS	0.041	0.980	0.992	0.997
STTR	**0.034**	**0.992**	**0.996**	**0.998**

Significant values are in bold.

### Experiment setup

The proposed framework was implemented on PyTorch. For STTR, the model^29^ that has been pre-trained in Scene Flow^38^ is used, and training is performed only for the monocular depth estimation model. The encoder is initialized with the DPT-Hybrid^27^ learned in advance, and the decoder is randomly initialized. Adam optimizer^39^ was used as the optimization algorithm with $\beta _1=0.9,\ \beta _2=0.999$. We set the encoder learning rate as $1.0\times {10}^{-5}$, and the decoder learning rate as $1.0\times {10}^{-4}$, both of which are exponentially attenuated with an attenuation factor of $\gamma =0.95$ for each epoch. The input images were flipped horizontally with a 50% probability, and they were also vertically flipped with a 50% probability. In addition, the data was expanded by adding Gaussian noise. The learning epoch was chosen as 20 with a batch size of 16. The learning of the proposed method was performed for each of the two types of weight functions, one with Hard Mask and the other with Soft Mask. In both cases, $\theta =0.5$ is set experimentally and the Soft Mask hyperparameter $\lambda =10$. These values were determined experimentally by grid search. We then compare the proposed method with conventional scale and shift-invariant methods that do not consider confidence. First, among those that train depth estimation models with scale and shift-invariant loss using depth ground truth as teaching data, the model trained with the loss function proposed by MiDaS^33^, which has shown the highest accuracy in recent years, is used for comparison. In endoscopic surgeries, where the camera moves extensively, and tasks like resection or suturing make acquiring the dense ground-truth depth difficult, adapting to varying features and biases is crucial. Therefore, MiDaS was selected as a comparative method due to its ability to handle these challenges. This comparison confirms the validity of the proposed method with and without confidence. Specifically, only the inverse depth provided by STTR was given as teaching data, and the same DPT-Hybrid as the proposed method was trained with the MiDaS loss function. All other conditions are the same as the proposed method. Second, among the SfMLearner methods that perform self-supervised learning of relative depth estimation models without depth ground truth as teaching data, we also compare AF-SfMLearner^24^, which is adapted to endoscopic environments and shows the highest accuracy compared to other SfMLearner methods for static endoscopic images of living subjects. The AF-SfMLearner was trained by using the original implementation published on GitHub. Finally, for the training data of this experiment, 80% of the 41,431 datasets provided as training data by Hamlyn Dataset, which is a dynamic data set, are used, and the remaining sets are used for validation.

### Evaluation on static datasets

**Figure 4:**
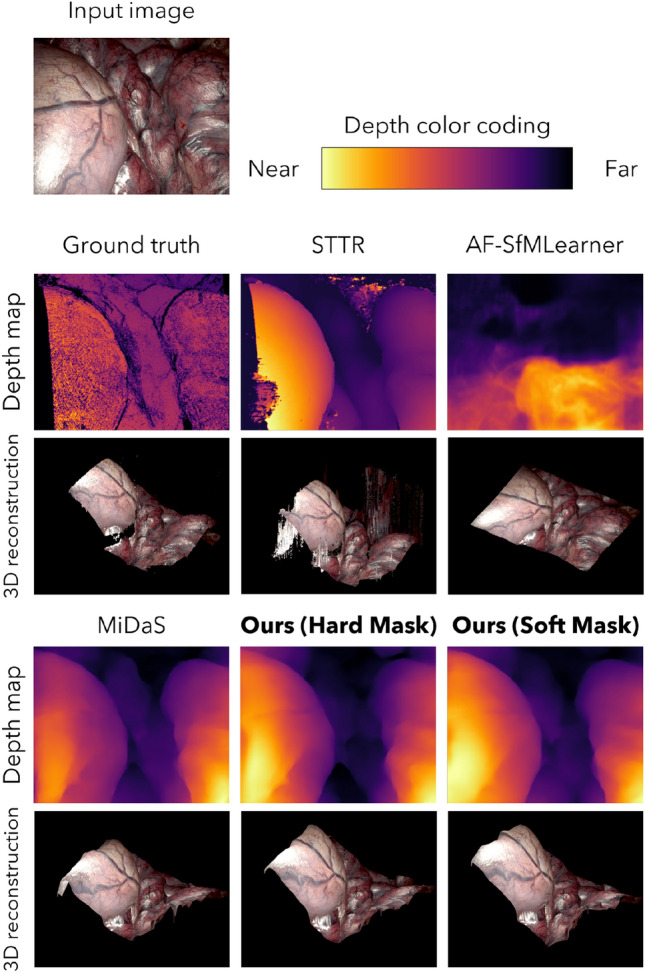
Visualization of predicted depth map and 3D reconstructed point cloud from a SCARED input image for each method. Note that we are using different datasets for the training and test data.

In order to directly evaluate the generalization performance of the proposed method and the accuracy of its depth estimation, depth estimation was done for the static dataset SCARED by using each method trained by Hamlyn Dataset, which is dynamic, and ground truth was directly evaluated quantitatively for estimation accuracy. In this instance, the evaluation was conducted following the IRLS alignment to the ground truth, as detailed in the evaluation index section. Note that we are using different datasets for the training and test data. As mentioned earlier, our objective is to perform depth estimation using real endoscopic images, such as those from the Hamlyn Dataset, which offers more diverse and extensive training data. This validation aimed to assess the depth estimation accuracy achievable with data trained based on the Hamlyn Dataset. To do so, we carried out depth estimation on the SCARED dataset using a depth estimator trained on the Hamlyn Dataset and evaluated the results. The results are shown in Table 2, which imply that the proposed method achieves the same depth estimation accuracy as the other latest monocular depth estimation methods for clear image input from static surgical fields without artifacts. Figure 4 shows an example of the predicted depth by each method and the resulting point cloud reconstructed three-dimensionally based on the predicted depth. The depth map is the one generated after alignment by IRLS. For the sake of convenience, the depth of invalid pixels is set to infinity.

**Table 2:** Quantitative evaluation of each model trained on Hamlyn Dataset, a dynamic dataset, against SCARED, a static dataset.

Method	Abs. Rel.	$\delta <1.25$	$\delta <1.25^2$	$\delta <1.25^3$
AF-SfMLearner	0.173	0.756	0.918	0.967
MiDaS	0.158	0.804	0.952	0.984
Ours (hard mask)	**0.156**	0.810	0.957	**0.986**
Ours (soft mask)	0.159	**0.829**	**0.958**	0.976

Note that we are using different datasets for the training and test data.

Significant values are in bold.

### Evaluation on dynamic datasets

**Figure 5:**
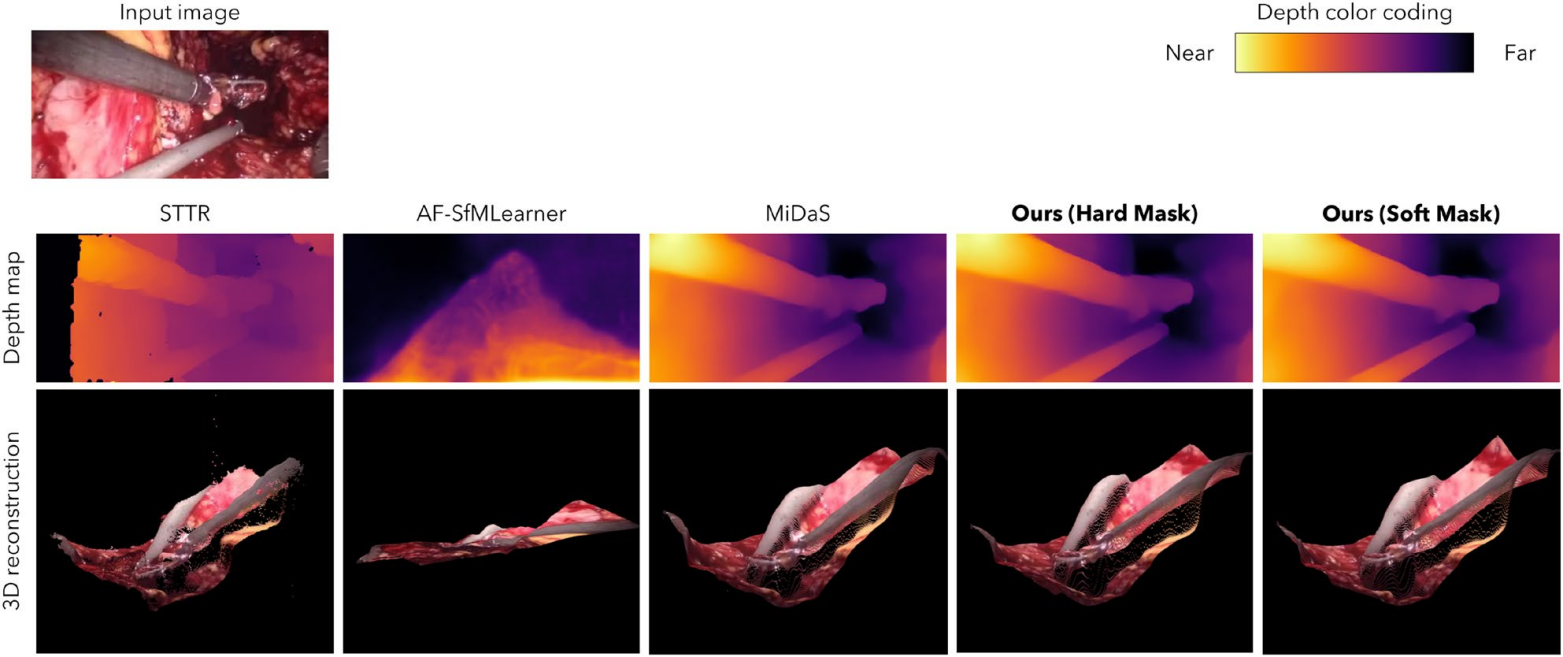
Visualization of predicted depth map and 3D reconstructed point cloud from a Hamlyn Dataset input image for each method.The outer diameter of fenestrated bipolar forceps is approximately 8.5 mm.

We evaluated the depth estimation accuracy of each method for laparoscopic scenes where the surgical field is dynamically deformed. Of the 7191 sets of sequence images for testing provided by Hamlyn Dataset, 100 sets were extracted at intervals of 70 images and evaluated by treating the calculated depth using STTR as indirect ground truth. Figure 5 shows an example of the predicted depth by each method and the point cloud reconstructed three-dimensionally based on it. Like SCARED, the depth map is the one after IRLS alignment, and the invalid pixel depth is set to infinity for convenience. In this study, as detailed in the evaluation index section, the indirect ground truth was evaluated post alignment using the IRLS method. The results, presented in Table 3, demonstrate that the proposed method offers the highest depth estimation accuracy for laparoscopic scenes characterized by dynamic deformation in the surgical field. Furthermore, the accuracy of the AF-SfMLearner is comparatively lower. This can be attributed to the fact that although learning takes place while masking the deformed and moving sections of the image using an explainability mask, a considerable portion of the image in this dataset is deformed and moving. This limits the improvement in accuracy due to masking, ultimately resulting in reduced learning accuracy. Therefore, the learning accuracy is deemed to be low. However, it is important to note that the AF-SfMLearner does not require stereo images for learning, which is beneficial when only monocular images are available. Moreover, effective training should be possible if a clean dataset can be prepared for areas with minimal deformation, such as the gastrointestinal tract. In fact, state-of-the-art results were achieved for static test data when training on a static dataset. Nevertheless, training on a dataset that captures the actual surgical environment, like the Hamlyn Dataset utilized in this study, which contains substantial noise such as deformations and artifacts, proves difficult and results in low accuracy. On the other hand, the proposed method that is trained by the disparity data and MiDaS are not affected in principle by the portion involving deformation and movement of the image, so relatively accurate learning is performed. In addition, it can be seen that the proposed method, which was trained by considering the confidence of the disparity information, realized higher depth estimation accuracy than the MiDaS, which was trained solely by the disparity information (Fig. S2). In Fig. S2, the outline of the rightmost surgical instrument is unclear in MiDaS, but the proposed method can render the instrument outline close to the actual endoscopic image. Furthermore, the results show that the Soft Mask, which increases loss weight for higher reliability, achieves higher accuracy than the Hard Mask. It is considered that the weighting according to the confidence information of the self-supervisory signal contributes to more efficient learning.

**Table 3:** Quantitative evaluation of each model against the Hamlyn Dataset test data.

Method	Abs. Rel.	$\delta <1.25$	$\delta <1.25^2$	$\delta <1.25^3$
AF-SfMLearner	0.701	0.401	0.652	0.798
MiDaS	0.254	0.757	0.906	0.948
Ours (hard mask)	0.236	0.776	0.917	**0.957**
Ours (soft mask)	**0.227**	**0.783**	**0.918**	0.955

Significant values are in bold.

## Discussion

The results demonstrated that monocular depth estimation on the SCARED test data, using models trained on the Hamlyn Dataset, can achieve estimation accuracy comparable to conventional methods. This indicates that a certain degree of accuracy in depth estimation can be obtained even when employing real endoscope images as the training dataset, as seen in the Hamlyn Dataset. Consequently, this enables large-scale learning using standard stereo endoscope images. The findings also suggest that the proposed method exhibits strong generalization performance and can attain depth estimation with reasonable accuracy, even when learning from different organ sites or laparoscopic cameras, or under varying surgical field conditions. Regarding the execution time required for inference, it takes 29.8 fps for the proposed method to perform depth estimation of the 384 × 192 image provided by Hamlyn Dataset with Intel Core i9-9900KF 3.6Ghz CPU and NVIDIA GeForce RTX 2080 Super GPU. This suggests the effectiveness of the proposed method in real-time applications such as surgical navigation and autonomous control of a surgical support robot.

In this paper, STTR was adopted as a module for handling self-supervisory signals with confidence information. When a novel stereo vision method emerges that may provide more accurate depth information with higher confidence and a novel hardware emerges that may provide such information without incurring any inconvenience even during surgery, they should be utilized as a module for self-supervisory signals with confidence information, to which the proposed method could be applied for learning to derive an improved monocular depth estimation model in a more accurate laparoscopic scene.

We have also realized highly accurate monocular depth estimation by incorporating confidence information into the learning even for image inputs from the Hamlyn Dataset that contain smoke and surgical instruments. This facilitates the acquisition of training datasets because the proposed method does not require the use of clean, static training datasets which are available only in a well-maintained environment. At the same time, the proposed method can directly use intraoperative laparoscopic scenes that might have been left out as a training dataset due to their poor quality such as noise, artifacts, and field deformation, so that larger scale learning with a wider range of data set may become feasible. Our proposed method has shown promise in being able to handle a variety of conditions, such as bleeding and smoke. However, it is limited in terms of the diversity of surgical scenes it can address. Therefore, further studies are needed to evaluate its usefulness in surgical fields where various surgical tools are used and in different types of surgeries beyond nephrectomy, such as pulmonary resection or hepatectomy and vascular anastomoses. Nevertheless, the advantage that actual intraoperative scenes can be used for learning suggests that the gap between the dataset used for learning and the image input obtained during real application-including differences in surgical tools, tissue types, surgical techniques, and individual variations-can be minimized. Based on these findings, the next steps will involve the construction of a comprehensive, large-scale dataset encompassing various surgical scenes to validate the generalizability of our approach further.

There are several issues yet to be solved for the proposed method. The first issue is the lack of quantitative evaluation of accurate ground truth for dynamic datasets because it is practically difficult to measure the exact depth of the ground truth for dynamically deformed objects in a body. This is not an issue peculiar to the proposed method, but is a universal problem encountered when evaluating depth estimation tasks in vivo^37^. Establishing a precise measurement method for the depth of tissues that are dynamically deformed in a living body is a major issue common to the field. Secondly, the estimation results of our framework provide relative depth information, and uncertainties in scale and shift are present. Firstly, when the depth estimated by our framework is used for surgical navigation or surgical robot control, it needs to be converted to the absolute scale of real space. This issue is also common among other self-supervised monocular depth estimation methods and multi-view stereo algorithms, such as SfM^17^. A separate application is necessary to recover the absolute scale, with potential approaches including the utilization of the camera’s internal parameters and scaling using the laparoscopic grasping arm’s internal parameters. Shift uncertainties also necessitate the identification of shift parameters using the laparoscope’s positional information via the robot and triangulation. Although the proposed method has the disadvantage of requiring a shift parameter recovery process, unlike methods that include shift parameters in learning, it is a scale-shift invariant model that can accurately estimate relative depth information across a wide range of shifts. This offers the advantage of increased generalizability, meaning that it can be applied clinically in various surgical environments, which is crucial for our method. In practical implementation of robot-assisted surgery or navigation, a critical issue is the conversion to absolute depth. Currently, our estimation is based on disparity. For future applications, it is conceivable to use methods such as extracting at least two feature points from the field of view based on the robot’s internal parameters, calculating their absolute depth, and then converting the entire field of view’s relative depth to absolute depth according to the camera model.

## Conclusion

In this study, we proposed a method for learning a dense monocular depth estimation model that employs a Vision Transformer as its backbone to utilize binocular disparity obtained from stereo images and its confidence information. The purpose of this method is to perform monocular depth estimation in dynamic laparoscopic scenes involving deformation and movement of the surgical field. By introducing a loss function that enables weighting and exclusion of self-monitoring signals based on confidence information, accurate depth estimation was achieved while utilizing actual surgical images as training datasets. This approach significantly lowers the hurdle in preparing the training dataset for depth estimation. The framework presented here is not only applicable to the depth estimation architecture proposed in this paper, but also to other methods where real surgical images can be employed as training data. The results of this study indicates the effectiveness and high generalization capability of the proposed method for image inputs with varying scene dynamics, laparoscopic procedures. Currently, the method provides relative depth rather than absolute depth. As a future challenge, it is necessary to integrate the scaling flow of the proposed method to a real-space scale. This integration is expected to lead to applications in autonomous control of surgical assist robots and surgical navigation. Since the ultimate users of our proposed method are humans, future work will need to consider not just the accuracy of the depth estimates but also factors such as the ease with which clinicians can interpret these estimates during surgery.

### Supplementary Information

Supplementary Figures.

## Data Availability

The datasets during and/or analyzed during the current study are available from the corresponding authors on reasonable request.
