# Supplementary material for: Confidence-aware self-supervised learning for dense monocular depth estimation in dynamic laparoscopic scene
Source: Sci Rep. 2023 Sep 16;13:15380. doi: 10.1038/s41598-023-42713-x (PMC10505201; doi:10.1038/s41598-023-42713-x)
Supplement: Supplementary file 1 — Supplementary Figures. [file 41598_2023_42713_MOESM1_ESM.pdf]

Supplementary Information

Confidence-aware self-supervised learning for dense monocular depth estimation in dynamic laparoscopic scene

Yasuhide Hirohata, Maina Sogabe*, Tetsuro Miyazaki, Toshihiro Kawase & Kenji Kawashima

Supplementary Figure

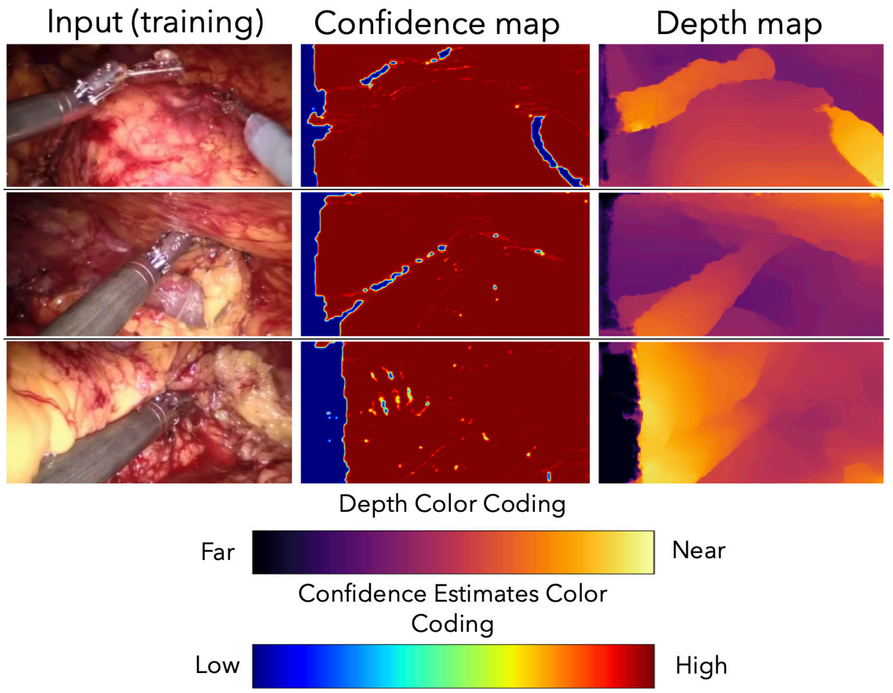

Figure S1. Confidence map and disparity results for the input image used for training data: Pixels on the left side of the left image used for the input image cannot be matched because the region is not present in the right side image and is judged as occlusion, resulting in the left side of the image having the confidence level of the left side of the input image is low. Also, the edges of the instruments cannot be matched due to the camera angle and other factors, resulting in a low confidence level (e.g., the surgical instrument in the lower right in the upper input image). The outer diameter of the fenestrated bipolar forceps is approximately 8.5 mm.

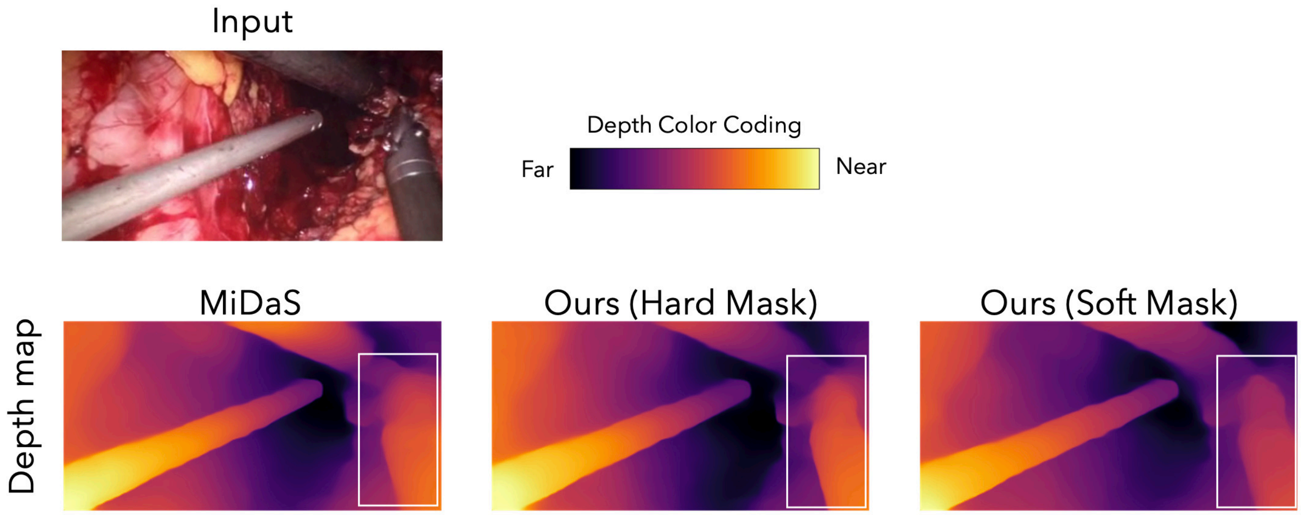

Figure S2. Comparison between MiDaS and the proposed method: The area of the lower right instrument, enclosed by a white rectangle, is overestimated by MiDaS, while the proposed method more accurately depicts the surgical instruments area. The outer diameter of the fenestrated bipolar forceps is approximately 8.5 mm.
